# Impact of intraoperative margin optimization strategies compared to standard breast-conserving surgery on oncologic outcomes: a systematic review and meta-analysis of randomized and prospective trials

**DOI:** 10.1186/s12957-025-03959-z

**Published:** 2025-08-27

**Authors:** Wajahat Mirza, Muhammad Moaz, Muhammad Sajeel Turab, Hadi Mohammad Khan, Sundus Dadan, Saeeda Yasmin, Abdullah Khan Tareen, Hamza Hanif

**Affiliations:** 1 Shifa College of Medicine, Shifa Tameer-e-Millat University, Sector H-8/4, Islamabad, Pakistan; 2 Department of General Surgery and Surgical Oncology, Shifa International Hospital, Islamabad, Pakistan; 3 Shifa’s Clinical Research Center, Shifa International Hospital, Islamabad, Pakistan; 4 Department of General Surgery, Shifa International Hospital, Islamabad, Pakistan

**Keywords:** Breast-conserving surgery, Intraoperative margin optimization, Positive margins, Re-excision rate, Local recurrence, Overall survival, Meta-analysis

## Abstract

**Background:**

Achieving optimal surgical margins is critical in breast-conserving surgery (BCS) to reduce local recurrence (LR) and the need for re-excision. This meta-analysis evaluated the impact of intraoperative margin optimization strategies on key surgical and oncologic outcomes in patients who underwent BCS.

**Methods:**

A systematic review and meta-analysis were conducted according to the PRISMA guidelines, including six randomized controlled trials (RCTs). The outcomes assessed included the re-excision rate (primary outcome), positive margin rate, local recurrence (LR), and overall survival (OS). The risk of bias was evaluated using the ROB 2 tool, and the certainty of evidence was assessed using GRADE. The study protocol was prospectively registered in the PROSPERO database (CRD420251000564).

**Results:**

Intraoperative margin optimization significantly reduced re-excision rates (OR 0.54, 95% CI 0.32–0.90), corresponding to 169 fewer re-excisions per 1,000 patients. Positive margin rates were also significantly lower (OR 0.40, 95% CI 0.22–0.73), translating to 139 fewer positive margins per 1,000 patients. No statistically significant differences were observed for LR (OR 0.72, 95% CI, 0.16–3.19) or OS (OR 0.87, 95% CI, 0.73–1.03).

**Conclusion:**

Intraoperative margin optimization effectively reduces positive margins and re-excisions in BCS without adversely affecting LR or OS. The incorporation of these strategies should be considered a standard practice to enhance surgical quality and patient outcomes.

**Supplementary Information:**

The online version contains supplementary material available at 10.1186/s12957-025-03959-z.

## Introduction

Breast-conserving surgery (BCS) followed by radiotherapy is the cornerstone of treatment for early-stage breast cancer, offering survival outcomes equivalent to those of mastectomy while preserving the breast [1]. However, achieving optimal surgical margins remains a critical determinant of local control. Inadequate margins are associated with an increased risk of ipsilateral breast tumor recurrence (IBTR), which can compromise cosmetic outcomes and, in some cases, lead to additional surgeries or the need for mastectomy [2]. Despite the adoption of “no tumor on ink” as the accepted standard for invasive disease in international guidelines [3], wide variations persist in surgical practice regarding margin optimization. Many surgeons continue to employ intraoperative techniques aimed at achieving wider margins or minimizing positive margin rates, particularly in patients with ductal carcinoma in situ (DCIS) or unfavorable tumor biology [4].

Several intraoperative margin optimization strategies have been developed to overcome this challenge. Techniques such as cavity shave margins (CSM), MarginProbe-assisted intraoperative assessment, and intraoperative radiotherapy (IORT) have gained traction as adjuncts to standard lumpectomy [5, 6]. These interventions aim to reduce the incidence of positive margins and the consequent need for re-excision, thereby improving patient experience and reducing the healthcare costs [7]. Importantly, while consensus guidelines provide clear recommendations for margin width in invasive carcinoma, the optimal intraoperative strategy to achieve these margins in a reproducible and efficient manner remains an area of active investigation [8].

Cavity shave margins, wherein additional tissue is excised circumferentially around the surgical cavity, have demonstrated reductions in positive margins and re-excision rates in several randomized trials [9]. MarginProbe, a radiofrequency spectroscopy device used intraoperatively to assess margin status, offers a technological adjunct to visual and tactile assessment, with the goal of reducing reoperation rates [10]. Meanwhile, IORT delivers a single high dose of radiation to the tumor bed intraoperatively, aiming to eradicate residual microscopic disease and serve as either a boost or definitive therapy [11]. Each of these strategies addresses the problem of margin adequacy from a different perspective—surgical, technological, or radiotherapeutic—highlighting the complexity of margin management in breast cancer care.

While individual studies have evaluated the efficacy of these approaches, the results have been heterogeneous, and no consensus has emerged regarding their comparative impact on surgical and oncologic outcomes [12]. Moreover, most prior analyses have focused on margin width as an isolated metric rather than examining the role of intraoperative optimization strategies as an integrated component of surgical planning. Given the potential implications for patient outcomes and healthcare resource utilization, a comprehensive synthesis of the available evidence is required.

Previous meta-analyses, such as that by Houssami et al. [2], have primarily examined the impact of margin width on local recurrence, often using retrospective or mixed designs. In contrast, our analysis uniquely focuses on intraoperative optimization strategies evaluated in randomized trials, capturing a more clinically actionable and strategy-driven perspective of the issue.

Therefore, we conducted a systematic review and meta-analysis of randomized and prospective trials to evaluate the impact of intraoperative margin optimization strategies on key surgical and oncologic outcomes of breast-conserving surgery. Our primary objective was to assess the effects of these interventions on re-excision rates. The secondary objectives included evaluation of positive margin rates, local recurrence, and overall survival. By synthesizing data across multiple strategies and surgical contexts, we aim to provide evidence to inform clinical decision-making and guide the integration of intraoperative margin management into routine clinical practice.

## Materials and methods

### Study design

This systematic review and meta-analysis adhered to the PRISMA 2020 guidelines. The completed PRISMA checklist is provided in the Supplementary Material. This study aimed to evaluate the impact of intraoperative margin optimization strategies on surgical and oncologic outcomes in patients undergoing breast-conserving surgery (BCS) for early-stage breast cancer. In this study, intraoperative margin optimization was defined as any strategy applied during the surgical procedure to improve surgical margin clearance, reduce positive margin rates, minimize the need for re-excision, and enhance local disease control. Strategies included within this definition were cavity shave margins, where additional tissue is excised circumferentially from the lumpectomy cavity; MarginProbe-assisted intraoperative margin assessment, a technological adjunct used to identify close or positive margins intraoperatively; and intraoperative radiotherapy (IORT), where a targeted dose of radiation is delivered directly to the tumor bed during surgery. Although whole breast radiotherapy (WBRT) is not typically considered an intraoperative intervention, data from the landmark Fisher 2002 trial were included in this analysis for long-term oncologic outcomes (local recurrence and overall survival) because of its historical relevance and the central role of radiotherapy in margin management during that era. The study protocol was prospectively designed and registered in the PROSPERO database registration number: [CRD420251000564].

### Literature search strategy

A comprehensive literature search was conducted across the PubMed, Embase, Cochrane Central Register of Controlled Trials (CENTRAL), and Scopus databases from database inception to May 2025. The search strategy combined terms and synonyms related to “breast-conserving surgery,” “lumpectomy,” “surgical margins,” “margin optimization,” “cavity shave margins,” “MarginProbe,” “intraoperative radiotherapy,” “local recurrence,” “re-excision,” and “positive margins.” The complete search strategy is provided in the Supplementary Material. In addition to electronic searches, the reference lists of all the included articles and relevant review articles were manually screened to identify any additional eligible studies. The complete, reproducible search strategy for each database is provided in Supplementary Table S1.

### Eligibility criteria

Studies were eligible for inclusion if they met the following criteria: randomized controlled trials (RCTs) or prospective cohort studies; enrolled adult patients (≥ 18 years) with stage 0–III breast cancer undergoing breast-conserving surgery; compared an intraoperative margin optimization strategy with standard breast-conserving surgery; and reported at least one of the predefined outcomes of interest: re-excision rate, positive margin rate, local recurrence, or overall survival. Additional inclusion criteria required that studies report a minimum median follow-up duration of 12 months and enroll at least 90 patients per arm to ensure adequate data reliability. Studies using historical control groups without a comparable arm, studies without extractable outcome data, and those conducted in the neoadjuvant setting were excluded. In the event of multiple publications from the same cohort, the most complete and updated dataset was selected.

### Data extraction and outcome measures

Data were independently extracted by two reviewers using a pre-designed, standardized form. Any discrepancies were resolved through discussion or consultation with a third reviewer, if necessary. The extracted information included study characteristics (first author, year of publication, country, and study design), patient demographics, tumor characteristics, sample size in experimental and control groups, type of margin optimization strategy, comparator intervention, duration of follow-up, and reported outcomes.

The primary outcome of this analysis was the re-excision rate, defined as the proportion of patients requiring an additional surgical procedure to achieve negative margins after the initial breast-conserving surgery. Secondary outcomes included the positive margin rate, defined as the proportion of patients with tumors present at the inked surgical margin or within 1 mm, as per the original study definitions, and local recurrence, defined as ipsilateral breast tumor recurrence occurring at any time during follow-up. Overall survival was analyzed as an exploratory outcome, defined as survival from any cause at the longest follow-up.

### Risk of bias assessment

Because this meta-analysis relied on published, peer-reviewed RCT data, no author contact was necessary. All outcomes were extracted from the full-text articles. The risk of bias of the included randomized controlled trials was independently assessed by two reviewers using the Cochrane Risk of Bias 2.0 (RoB 2) tool. The tool evaluates five domains: randomization process, deviations from intended interventions, missing outcome data, measurement of outcomes, and selection of reported results. Discrepancies between the reviewers were resolved by consensus. Risk of bias assessments informed the interpretation of the meta-analysis findings.

### Statistical analysis

Meta-analyses were performed using Review Manager (RevMan) version 5.4 (The Cochrane Collaboration). For all outcomes, a random-effects model was employed using the DerSimonian and Laird method, which accounts for both within-study and between-study variability and is considered more conservative when clinical heterogeneity is anticipated across the studies. Risk ratios (RRs) with corresponding 95% confidence intervals (CIs) were calculated for dichotomous outcomes.

Statistical heterogeneity across studies was quantified using the I² statistic, with I² values of 25%, 50%, and 75% interpreted as low, moderate, and high heterogeneity, respectively, respectively. Given the small number of studies contributing to each outcome (< 10), a formal assessment of publication bias through funnel plot analysis was not performed, following the Cochrane guidance. All statistical tests were conducted using a two-sided alpha level of 0.05, with statistical significance defined as such.

### Certainty of evidence, sensitivity analyses, and subgroup analyses

The certainty of evidence for each outcome was evaluated using the Grading of Recommendations Assessment, Development, and Evaluation (GRADE) approach. This process considered factors such as the risk of bias, inconsistency, indirectness, imprecision, and potential publication bias. Each outcome was rated as having high, moderate, low, or very low certainty of evidence, based on this framework.

Sensitivity analyses were conducted to explore the robustness of the primary findings by systematically excluding individual studies and rerunning the meta-analysis to assess the stability of the pooled estimates. Subgroup analyses were pre-specified to explore the potential sources of heterogeneity. In the case of substantial heterogeneity in any outcome, subgroup analyses were planned based on the duration of follow-up, categorized as short-term (≤ 3 years) or long-term (> 3 years), according to the median follow-up reported in the included studies. This approach was selected to assess whether the follow-up duration influenced surgical or oncologic outcomes.

## Results

### Study characteristics of included articles

**Fig. 1:**
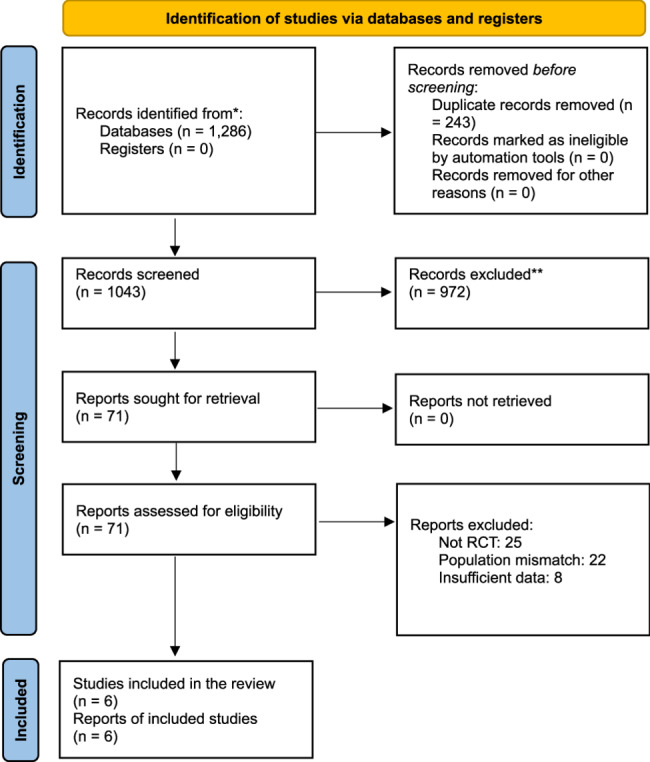
PRISMA 2020 flow diagram illustrating the study selection process

A total of 1,286 records were identified through the database search. After the removal of 243 duplicate records, 1,043 records remained for title and abstract screening. Of these, 972 records were excluded based on their relevance to the study question. A full-text review was conducted for 71 articles, with 65 subsequently excluded for reasons including wrong population (*n* = 25), no eligible control group (*n* = 22), no extractable outcome data (*n* = 10), or ineligible study design (*n* = 8). Ultimately, six randomized controlled trials were included in this meta-analysis. The study selection process is illustrated in Fig. 1.

The six included studies comprised a total of 4,968 patients, with 2,471 in the experimental group and 2,497 in the control group. The studies were conducted in the United States and the United Kingdom, with publication dates ranging from 2002 to 2020. The evaluated intraoperative margin optimization strategies included cavity shave margins, MarginProbe-assisted lumpectomy, and intraoperative radiotherapy. The control group received standard breast-conserving surgery with or without whole-breast radiotherapy. Follow-up durations ranged from 12 months to 20 years, allowing for the assessment of both short-term surgical outcomes and long-term oncologic endpoints. A summary of the key study characteristics is provided in Table 1.

**Table 1:** Characteristics of included studies evaluating intraoperative margin optimization strategies in Breast-Conserving surgery

Study (First Author, Year)	Country	Study Design	Sample Size (Exp/Ctrl)	Experimental (Intraoperative Margin Optimization Strategies)	Control Group (Standard Breast-Conserving Surgery)	Inclusion Criteria	Exclusion Criteria	Primary Outcomes Reported	Follow-Up Duration
Schnabel, 2014 [13]	USA	RCT	298/298	MarginProbe-assisted lumpectomy	Standard lumpectomy	Early-stage breast cancer (Stage 0-II), non-palpable tumors	Neoadjuvant chemotherapy, previous ipsilateral RT	Positive margin rate, re-excision rate	Median 12 months
Vaidya, 2020 [14]	UK	RCT	1140/1158	Intraoperative radiotherapy (IORT)	Postoperative WBRT	Women ≥ 45 years with invasive breast cancer are suitable for BCS	Multicentric disease, prior ipsilateral breast cancer, pregnancy	Local recurrence, overall survival	Median 8.6 years
Chen, 2019 [15]	USA	RCT	90/91	Cavity shave margins	No cavity shave margins	Patients ≥ 18 years with stage 0-III breast cancer undergoing lumpectomy	Prior ipsilateral breast cancer, neoadjuvant chemotherapy	Positive margin rate, re-excision rate	Median 12 months
Fisher, 2002 [16]	USA	RCT	628/634	Lumpectomy + WBRT	Lumpectomy alone	Stage I-II breast cancer, tumor ≤ 4 cm, clinically node-negative	Previous breast cancer, prior RT, neoadjuvant therapy	Local recurrence, overall survival	Median 20 years
Dupont, 2019 [17]	USA	RCT	196/200	Cavity shave margins	No cavity shave margins	Stage 0-III breast cancer undergoing BCS	Neoadjuvant chemotherapy, bilateral breast cancer	Positive margin rate, re-excision rate	Median 12 months
Chagpar, 2015 [18]	USA	RCT	119/116	Cavity shave margins	No cavity shave margins	Stage 0-III breast cancer, BCS planned	Neoadjuvant chemotherapy, bilateral breast cancer	Positive margin rate, re-excision rate, and local recurrence	Median 12 months

### Patient demographics, clinical characteristics, and tumor characteristics

The patient demographics, clinical characteristics, and tumor characteristics of the included studies are summarized in Table 2. The mean or median age of the patients ranged from 58 to 65 years. Most patients had stage 0 to II breast cancer, with both invasive carcinoma and ductal carcinoma in situ represented in all studies. Hormone receptor-positive disease predominated, with HER2-positive tumors comprising a minority of cases. The median tumor sizes were generally small, ranging from 1 to 1.5 cm. The reporting of tumor grade, nodal status, and lymphovascular invasion varied across studies, reflecting the clinical heterogeneity of the included populations.

**Table 2:** Patient demographics, clinical characteristics, tumor characteristics, and stage distribution of included studies

Study	Median/Mean Age (years)	Histology (%)	ER Positive (%)	PR Positive (%)	HER2 Positive (%)	Median/Mean Tumor Size (cm)	High Grade (%)	Node Positive (%)	Stage 0 (*n*)	Stage I (*n*)	Stage II (*n*)	Stage III (*n*)
Schnabel, 2014 [13]	Mean 61	Invasive + DCIS	NR	NR	NR	NR	NR	NR	NR	NR	NR	NR
Vaidya, 2020 [14]	Mean 62.9	Invasive	80.6%	69.8%	17.4%	Median 1.2	12%	22%	NR	NR	NR	NR
Chen, 2019 [15]	Median 63	Invasive + DCIS	84%	76%	15%	Median 1.3	15%	21%	5	89	69	18
Fisher, 2002 [16]	Median 61	Invasive	78%	66%	13%	Median 1.5	14%	27%	NR	NR	NR	NR
Dupont, 2019 [17]	Median 65	Invasive + DCIS	84%	74%	11%	NR	NR	NR	71	279	68	9
Chagpar, 2015 [18]	Median 61	Invasive + DCIS	85%	76%	16%	Median 1.1	10%	18%	56	122	43	3

### Risk of bias

**Fig. 2:**
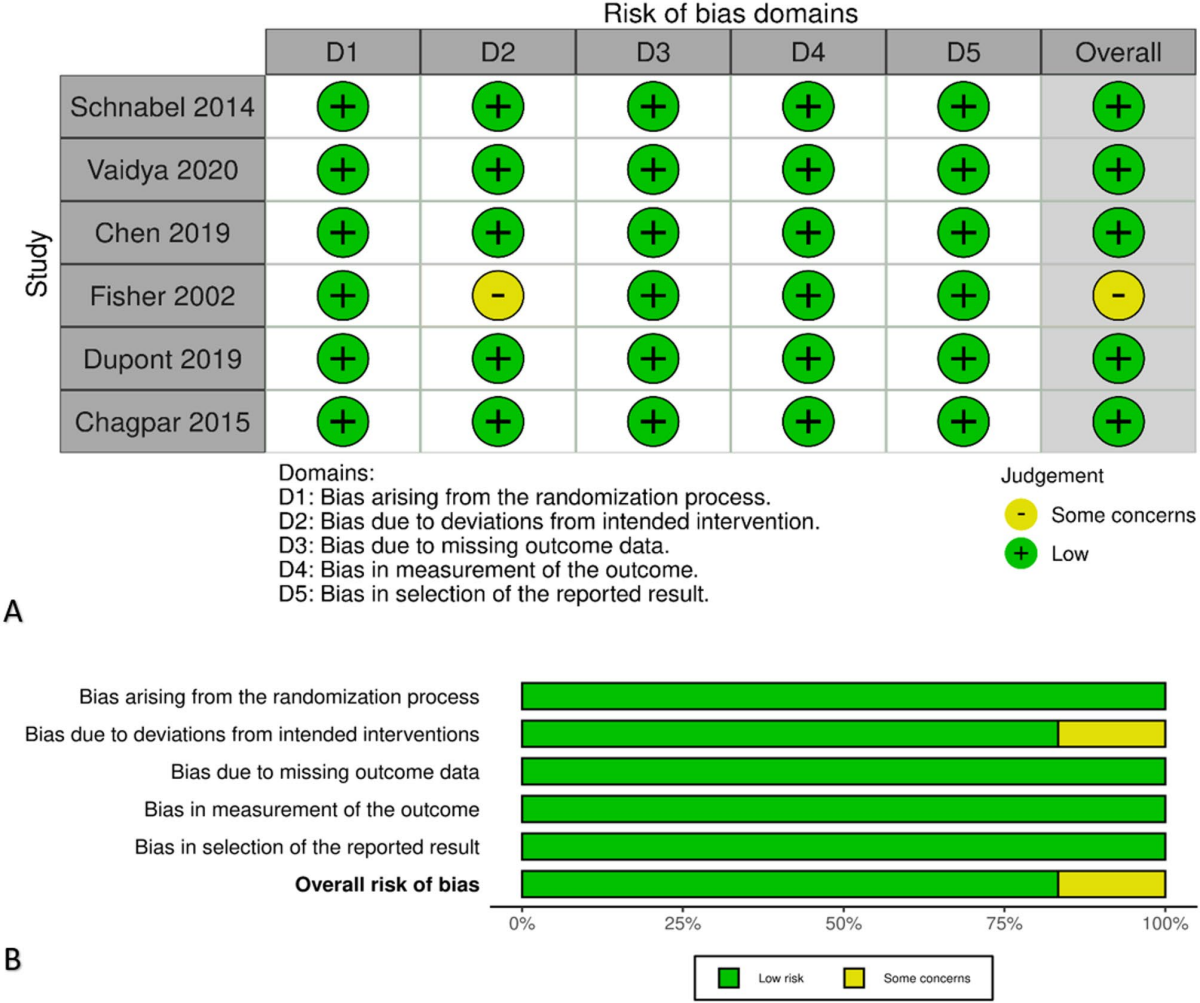
Risk of bias assessment of the included randomized trials (Cochrane RoB 2). **A** Traffic‑light plot showing domain‑level judgments for each trial. **B** Summary bar plot showing the proportion of trials rated at each risk level by domain

Risk of bias was assessed using the Cochrane RoB 2 tool. Overall, the included randomized controlled trials demonstrated a low risk of bias across most domains. One study (Fisher 2002) was judged to have some concerns regarding deviations from the intended interventions, resulting in an overall judgment of some concerns. The remaining studies were rated as having a low risk of bias in all domains. The distribution of the risk of bias judgments is summarized in Fig. 2.

#### Primary outcome

##### Re-excision rate

**Fig. 3:**
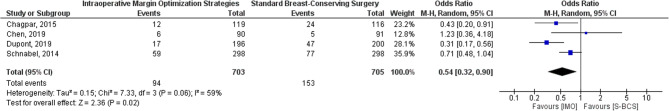
Forest plot of the effect of intraoperative margin optimization versus standard breast-conserving surgery on re-excision rate

Intraoperative margin optimization strategies resulted in a significantly reduced re-excision rate compared to standard breast-conserving surgery (OR 0.54, 95% CI 0.32–0.90, *p* = 0.02), with moderate heterogeneity (I² = 59%) as seen in Fig. 3.

#### Secondary outcomes

##### Positive margin rate

**Fig. 4:**
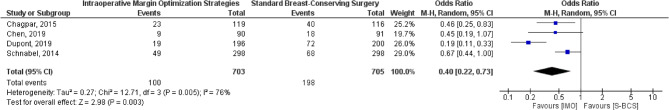
Forest plot of the effect of intraoperative margin optimization versus standard breast-conserving surgery on positive margin rate

Intraoperative margin optimization strategies were associated with a significantly lower positive margin rate than standard breast-conserving surgery (OR 0.40, 95% CI 0.22–0.73, *p* = 0.003), although substantial heterogeneity was present (I² = 76%) as illustrated in Fig. 4.

##### Local recurrence

**Fig. 5:**
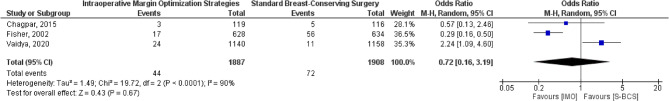
Forest plot of the effect of intraoperative margin optimization versus standard breast-conserving surgery on local recurrence

No statistically significant difference in local recurrence was observed between intraoperative margin optimization strategies and standard breast-conserving surgery (OR 0.72, 95% CI 0.16–3.19, *p* = 0.67). Considerable heterogeneity was noted (I² = 90%) as depicted in Fig. 5.

### Exploratory outcome

#### Overall survival

**Fig. 6:**
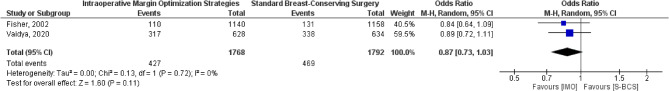
Forest plot of the effect of intraoperative margin optimization versus standard breast-conserving surgery on overall survival

There was no statistically significant difference in overall survival between patients undergoing intraoperative margin optimization and those treated with standard breast-conserving surgery (OR 0.87, 95% CI 0.73–1.03, *p* = 0.11), with no observed heterogeneity (I² = 0%) as presented in Fig. 6.

In this meta-analysis, intraoperative margin optimization strategies significantly reduced both re-excision and positive margin rates compared to standard breast-conserving surgery. However, no significant differences were observed in the local recurrence or overall survival outcomes. These findings suggest a clear surgical benefit of intraoperative optimization, although its long-term oncologic impact requires further investigation.

#### Sensitivity analysis

Sensitivity analyses for positive margin rate and local recurrence demonstrated that the overall effect estimates remained consistent upon exclusion of individual studies. Notably, heterogeneity dropped to 0% when Dupont 2019 and Vaidya 2020 were excluded from the analyses. These findings confirm the robustness of pooled outcomes despite the initial heterogeneity (Table 3).

**Table 3:** Sensitivity analysis showing the effect of excluding individual trials on pooled outcomes and heterogeneity (I²)

Outcome	Effect Estimate (Before)	*P*-value (Before)	I² % (Before)	Study Removed	Effect Estimate (After)	*P*-value (After)	I² % (After)
Positive Margin Rate	0.40 [0.22, 0.73]	0.003	76%	Chagpar 2015	0.39 [0.17, 0.90]	0.03	84%
Positive Margin Rate	0.40 [0.22, 0.73]	0.003	76%	Chen 2019	0.39 [0.19, 0.83]	0.01	84%
Positive Margin Rate	0.40 [0.22, 0.73]	0.003	76%	Dupont 2019	0.57 [0.42, 0.78]	0.0004	0%
Positive Margin Rate	0.40 [0.22, 0.73]	0.003	76%	Schnabel 2014	0.33 [0.18, 0.61]	0.0004	62%
Local Recurrence	0.72 [0.16, 3.19]	0.67	90%	Chagpar 2015	0.79 [0.11, 5.94]	0.82	95%
Local Recurrence	0.72 [0.16, 3.19]	0.67	90%	Fisher 2002	1.32 [0.36, 4.86]	0.67	63%
Local Recurrence	0.72 [0.16, 3.19]	0.67	90%	Vaidya 2020	0.31 [0.19, 0.53]	0.0001	0%

#### GRADE assessment

Using the GRADE approach, the certainty of evidence was rated as moderate for the re-excision rate and positive margin rate, low for local recurrence due to substantial heterogeneity and imprecision, and high for overall survival given consistent and precise estimates. The absolute effects ranged from 169 fewer re-excisions to 20 fewer deaths per 1,000 patients treated with intraoperative margin optimization strategies (Table 4).

**Table 4:** Summary of GRADE certainty of evidence assessment for primary, secondary, and exploratory outcomes

Outcome	Risk of Bias	Inconsistency	Indirectness	Imprecision	Publication Bias	Effect Estimate	Absolute Effect	GRADE Certainty
Re-excision Rate	Low	Moderate (I² = 59%)	None	None	None	OR 0.54 [0.32–0.90]	169 fewer per 1,000	Moderate
Positive Margin Rate	Low	Substantial (I² = 76%)	None	None	None	OR 0.40 [0.22–0.73]	139 fewer per 1,000	Moderate
Local Recurrence	Low	Very high (I² = 90%)	None	Serious (wide CI)	None	OR 0.72 [0.16–3.19]	14 fewer per 1,000	Low
Overall Survival	Low	None (I² = 0%)	None	None	None	OR 0.87 [0.73–1.03]	20 fewer per 1,000	High

#### Subgroup analysis

Although subgroup analysis based on the duration of follow-up was initially planned to explore the sources of heterogeneity, insufficient numbers of studies within each subgroup prevented meaningful stratification for both positive margin rate and local recurrence. Specifically, no long-term studies were available for positive margin rates, and too few studies reported local recurrence to support a valid subgroup analysis.

## Discussion

Breast-conserving surgery (BCS) combined with adjuvant whole-breast radiotherapy (WBRT) has long been established as an oncologically safe and effective treatment for early stage breast cancer [19, 20]. However, achieving optimal surgical margins remains a critical determinant of local control, cosmetic outcomes, and the need for re-excision [1, 2]. The present meta-analysis, which included six randomized controlled trials encompassing a substantial patient cohort, provides contemporary evidence on the efficacy of intraoperative margin optimization strategies in improving surgical and oncologic outcomes in BCS.

Our findings demonstrated that intraoperative margin optimization significantly reduced both positive margin and re-excision rates compared to standard BCS, without compromising local recurrence or overall survival. The 169 fewer re-excisions and 139 fewer positive margins per 1,000 patients highlighted a tangible clinical benefit. These results are consistent with those of prior meta-analyses showing that wider or more rigorously assessed margins are associated with improved local control [2]. Importantly, while our results support margin optimization, they also align with the established understanding that margins wider than “no ink on tumor” do not confer additional benefit when adequate radiotherapy is delivered, as underscored by the SSO-ASTRO consensus guideline [21].

The significant reduction in the positive margin rate corroborates earlier findings from observational studies and meta-analyses, suggesting that close or involved margins increase the risk of local recurrence [1–4]. In particular, studies by Gage et al. [1] and Houssami et al. [2] emphasized the prognostic relevance of margin status in BCS. Our analysis, drawing on more recent randomized controlled data, strengthens the evidence that intraoperative strategies, including cavity shave margins [18], use of devices such as MarginProbe [13], and targeted intraoperative radiotherapy [14], can meaningfully reduce positive margin rates, thereby sparing patients from additional surgery.

The observed reduction in re-excision rates is clinically significant, given the well-documented physical, psychological, and economic burdens associated with reoperations [5, 6]. Our findings resonate with those of a prospective trial by Chagpar et al. [18], which demonstrated a halving of re-excision rates with cavity shave margins. Similarly, the randomized trial by Dupont et al. [17] and the results of MarginProbe studies [13, 22, 23] highlight that technological adjuncts and systematic margin assessment can reduce re-excision rates without compromising cosmetic outcomes.

Although local recurrence did not differ significantly between the groups, the absolute risk reduction of 14 fewer local recurrences per 1,000 patients remained clinically relevant. This finding mirrors observations from long-term studies, indicating that meticulous surgical technique combined with modern radiotherapy minimizes local failure [19, 20, 24, 25]. Importantly, our results align with the SSO-ASTRO consensus [21] and trials such as Fisher et al. [16] and Vaidya et al. [14], which have shown excellent local control with optimized BCS approaches. The lack of a significant difference in overall survival aligns with the broader literature, affirming that local control improvements do not necessarily translate into survival gains when effective systemic therapy is provided [16, 20, 26, 27].

Our findings must be contextualized within the evolving landscape of breast cancer surgery in the future. While wider excisions may improve margin status, excessive tissue removal can compromise cosmetic outcomes, particularly in small breasts [28–30]. Intraoperative optimization strategies offer a balanced approach, enabling high rates of margin clearance with minimal tissue excision. Moreover, techniques such as targeted intraoperative radiotherapy [9–12, 14] and real-time margin assessment devices [7, 8, 13] continue to evolve, providing surgeons with increasingly sophisticated tools to achieve optimal oncologic and aesthetic outcomes in the future. However, it is important to note that this meta-analysis did not directly compare individual margin optimization strategies, such as cavity shave margins, MarginProbe-assisted assessment, or intraoperative radiotherapy. Rather, the current evidence supports the general effectiveness of intraoperative margin optimization as an intervention class. The comparative efficacy and cost-effectiveness of specific strategies remain important areas for future investigation. Additionally, the cost implications of these interventions, particularly for MarginProbe and intraoperative radiotherapy, should be carefully considered, as they may influence clinical adoption and health system decision-making.

Unlike earlier studies that focused on margin width alone [2], our review integrates evidence from randomized controlled trials specifically assessing intraoperative techniques—MarginProbe, cavity shave margins, and intraoperative radiotherapy—thereby offering higher methodological rigor and contemporary clinical relevance.

While this meta-analysis was sufficiently powered to detect differences in surgical outcomes, such as re-excision and positive margin rates, it may be underpowered for oncologic endpoints, such as local recurrence and overall survival, due to low event rates and wide confidence intervals. Therefore, these findings should be interpreted with caution. Larger long-term RCTs or individual patient-level data meta-analyses are warranted to provide more definitive estimates of these outcomes.

In conclusion, this meta-analysis provides robust evidence that intraoperative margin optimization significantly reduces positive margins and re-excisions in BCS, without adversely affecting local recurrence or overall survival. These findings support the incorporation of systematic margin assessment and optimization into routine surgical practice, consistent with the guideline recommendations [21, 31, 32]. Future research should continue to refine these techniques and explore their integration with emerging technologies to further enhance the quality of breast-conserving therapies.

## Conclusion

Intraoperative margin optimization is a valuable advancement in the surgical management of early-stage breast cancer. This meta-analysis of contemporary randomized controlled trials demonstrates that such strategies can substantially lower positive margin rates and the need for re-excisions without compromising local recurrence or overall survival. These findings reinforce the role of systematic intraoperative margin assessment as an integral component of breast-conserving surgery, supporting its routine implementation in clinical practice and improving patient outcomes. As surgical techniques and technologies continue to evolve, ongoing research should aim to further refine these approaches and directly compare different optimization strategies through head-to-head trials to ensure optimal oncologic outcomes while preserving cosmetic integrity and patient quality of life.

### Limitations

This meta-analysis has several limitations. First, the number of included randomized trials was modest, and some outcomes were inconsistently reported, limiting the ability to conduct subgroup analyses. Second, substantial heterogeneity in outcomes, such as positive margin rate and local recurrence, likely reflects differences in surgical techniques, definitions of margin status, and follow-up duration. Although sensitivity analyses confirmed the robustness of the key findings, these variations may have affected generalizability. Third, although intraoperative margin optimization was broadly effective, differences in techniques, such as cavity shave margins, intraoperative radiotherapy, and MarginProbe, limited direct comparisons between specific methods.

The radiotherapy protocols varied among the included studies, with some using whole-breast radiotherapy (WBRT) and others employing intraoperative radiotherapy (IORT). This variation reflects real-world clinical practice but introduces potential heterogeneity in the local recurrence outcomes. Owing to limited subgroup data, stratified analysis by radiation modality was not feasible, although this remains a recognized limitation. Additionally, while most studies enrolled patients with both invasive carcinoma and ductal carcinoma in situ (DCIS), the outcomes were not consistently reported by histologic subtype. Therefore, although our findings are broadly applicable, the specific impact of intraoperative margin optimization in DCIS warrants further investigation.

Finally, as with all meta-analyses, the potential for unrecognized publication bias cannot be entirely excluded despite the rigorous search strategy employed. Moreover, owing to the limited number of studies included per outcome (< 10), a formal assessment of publication bias using funnel plots was not performed in accordance with the Cochrane guidelines.

## Supplementary Information

Supplementary Material 1.

Supplementary Material 2.

## Data Availability

No datasets were generated or analysed during the current study.

## Abbreviations

BCS: Breast—conserving surgery
CI: Confidence interval
IORT: Intraoperative radiotherapy
LR: Local recurrence
OR: Odds ratio
OS: Overall survival
RCT: Randomized controlled trial
ROB 2: Risk of Bias 2 tool
SSO: ASTRO—Society of Surgical Oncology—American Society for Radiation Oncology
WBRT: Whole—breast radiotherapy
