# Supplementary material for: Impact of intraoperative margin optimization strategies compared to standard breast-conserving surgery on oncologic outcomes: a systematic review and meta-analysis of randomized and prospective trials
Source: World J Surg Oncol. 2025 Aug 27;23:322. doi: 10.1186/s12957-025-03959-z (PMC12382061; doi:10.1186/s12957-025-03959-z)
Supplement: Supplementary file 1 — Supplementary Material 1. [file 12957_2025_3959_MOESM1_ESM.docx]

**Supplementary Table S1. Reproducible Search Strategies for Each Database**

| **Database** | **Search Strategy** | **Date of Last Search** |
| --- | --- | --- |
| **PubMed** | ("breast-conserving surgery"[MeSH Terms] OR "lumpectomy"[All Fields]) AND ("surgical margins"[MeSH Terms] OR "margin optimization"[All Fields] OR "positive margins"[All Fields] OR "intraoperative margin assessment"[All Fields]) AND ("randomized controlled trial"[Publication Type] OR "prospective study"[All Fields]) | May 20, 2025 |
| **Embase** | ('breast conserving surgery'/exp OR 'lumpectomy':ti,ab) AND ('surgical margin'/exp OR 'positive margins':ti,ab OR 'margin optimization':ti,ab OR 'intraoperative margin assessment':ti,ab) AND ('randomized controlled trial'/exp OR 'prospective study'/exp OR 'clinical study'/exp) | May 20, 2025 |
| **Scopus** | TITLE-ABS("breast-conserving surgery" OR "lumpectomy") AND TITLE-ABS("margin optimization" OR "positive margins" OR "intraoperative assessment") AND TITLE-ABS("randomized controlled trial" OR "RCT" OR "prospective study") AND (LIMIT-TO(DOCTYPE, "ar") AND LIMIT-TO(SUBJAREA, "MEDI")) | May 20, 2025 |
| **Cochrane CENTRAL** | ("breast cancer" AND "breast-conserving surgery" AND "margin optimization" AND "intraoperative" AND ("randomized controlled trial" OR "prospective study")):ti,ab,kw | May 20, 2025 |
